# From the Destruction of Two Lumbar Segments to Thoracic‐Lumbar‐Pelvic Fusion: A Case Caused by Congenital Insensitivity to Pain with Anhidrosis and Literature Review

**DOI:** 10.1111/os.13746

**Published:** 2023-05-08

**Authors:** Yuhao Jiao, Ye Tian, Siyi Cai

**Affiliations:** ^1^ Department of Medicine Peking Union Medical College Hospital, Chinese Academy of Medical Sciences and Peking Union Medical College Beijing China; ^2^ Department of Orthopaedics Peking Union Medical College Hospital, Chinese Academy of Medical Sciences and Peking Union Medical College Beijing China

**Keywords:** Complications, Congenital insensitivity to pain with anhidrosis, NTRK1, Revision surgeries

## Abstract

**Background:**

Congenital insensitivity to pain with anhidrosis (CIPA) with Charcot arthropathy is a rare combination in orthopaedic clinical practice. The experience dealing with such patients is limited. Here with this case of approximately 10 years follow‐up, we wish to shed light on the choices of strategies of surgeries and alerting clinicians with post‐surgery complications. The possible underlying reasons for the recurrent Charcot arthropathies as well as strategies for peri‐operative management for such surgical cases are also discussed.

**Case Presentation:**

The patient underwent a surgery to correct her severe kyphosis caused by CIPA‐related Charcot spine. Multiple post‐surgery complications occurred during her follow‐up, including hardware migration, adjacent segment disease (ASD), and loosening pedicle screws. Five revision surgeries were conducted consequently. From the limited experience on the management of CIPA‐related Charcot spine, surgical correction is still the first‐line treatment.

**Conclusions:**

Of all the 16 cases reviewed (including our case), loosening pedicle screws, hardware migration, and ASDs are the common post‐surgery complications. Large‐scale removal of damaged vertebrae and subsequent reconstruction are not recommended, which might increase the risk of hardware migration. A 360° long‐segment fusion might be of help to reduce the risk of ASDs. In the meantime, comprehensive management including careful nursing, proper rehabilitation exercises, and treatments targeting bone mineral metabolism is also critical.

## Graphical abstract

Congenital insensitivity to pain with anhidrosis (CIPA) is a rare disease that can cause Charcot arthropathy and lead to spine deformity. The patient underwent a series of surgeries to correct her severe kyphosis and multiple post‐surgery complications, eventually resulting in a thoracic‐lumbar‐pelvic fusion. The article reviews related reports and wish to share our experiences on dealing with future cases.

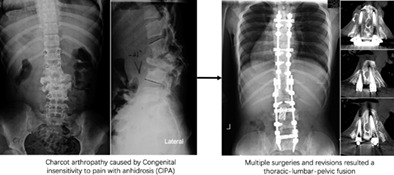

## Introduction

Congenital insensitivity to pain with anhidrosis (CIPA) is a rare and heritable autosomal recessive condition that is characterized by the lack of pain perception and absence of perspiration. The autosomal recessive condition is caused by loss‐of‐function mutations of the neurotrophic receptor tyrosine kinase 1 (NTRK1) gene, also known as tropomyosin receptor kinase A (TRKA) gene, which encodes a receptor for nerve growth factor (NGF).^1^ Typical clinical manifestations of CIPA include the loss of sensation to pain and temperature, anhidrosis, and intellectual deficit, which are directly associated with impairments of the nervous system.^2^ Hyperpyrexia is common and remains the predominant cause of death during childhood.^3^, ^4^ Apart from neurological disorders, spinal lesions and arthrosis affecting other joints can also occur in CIPA patients.^4^ These orthopaedic manifestations have largely been attributed to Charcot arthropathy. Several cases of CIPA patients with Charcot spine have been reported across the globe and the typical treatment for these patients is correction surgery. However, long‐term follow‐up studies on the recovery of CIPA patients with Charcot spine post‐surgery are currently lacking. Here, we report our observations on of a CIPA patient with recurrent Charcot arthropathies in her thoracic‐lumbar spine who had undergone five revision surgeries between 2010 to 2016, detailing multiple post‐surgery complications. The possible underlying reasons for the recurrence of her Charcot arthropathies as well as the strategy for managing such surgical cases are also discussed herein.

## Case Presentation

The patient is a Chinese female of Han nationality who was born in 1995. The course of disease and treatments of the patient are summarized in Table 1. At 11 months old, the patient developed high fever with unknown cause and was unable to perspire. Neurological examinations and skin biopsy confirmed the patient's insensitivity to superficial pain stimuli and a positive iodine‐starch test revealed anhidrosis. She was then diagnosed with congenital insensitivity to pain with anhidrosis (CIPA) due to an insertion mutation at exon 8 and a missense mutation at exon 10 of the tyrosine kinase receptor type 1 (NTRK1) gene. Between 1997 and 2000, the patient suffered recurrent bone fractures, including three right lower limb fractures, three left lower limb fractures, and a left femoral shaft fracture. Each fracture was treated by either plaster immobilization or surgical internal fixation at the local hospital. Multiple fractures and surgical corrections resulted in a discrepancy between lower limb lengths, with the right being shorter than the left.

**TABLE 1:** Course of disease and treatments of the CIPA case

Age	Year	Complications from Previous Treatments	Clinical Manifestations	Physical Examination	Imaging	Diagnosis	Surgical Treatment	Post‐surgery Management/Recovery
11 m.o.	1996		Fever, anhidrosis	Insensitivity to pain		CIPA		
				Iodine‐starch test positive				
2–5 y.o.	1997–2000		3 right crus fractures;	N.A.		Recurrent bone fractures	Plaster immobilization, surgical treatment	
			3 left crus fractures;					
			1 left femoral shaft fracture					
5 y.o.	2000	Shortening of right lower limb	Worsening muscular weakness	N.A.		Lumbar kyphosis		
15 y.o.	JAN/2010			Dry skin	lumber kyphosis deformity	CIPA	Posterior corpectomy of L2, L3	Immobilization in a thoracolumbosacral orthosis for 3 months
				increased motions of lumbar	Charcot arthropathies, destruction in centrums from L1 to L4	Charcot arthropathy	+ Mesh cage with autologous bone transplant	
				Shortened right lower limb	Compression of dual sac	Lumbar kyphosis	+ Fusion and instrumentation with pedicle screw from T12 to L5	
				Grade 4/5 muscular strength of right quadriceps and anterior tibialis		Lumbar canal stenosis		
15 y.o.	AUG/2010	Loosening pedicle screws		Grade 4/5 muscular strength of anterior tibialis			Revision:	Muscular strength fully recovered
							Reinforcement of the broken pedicle walls by allo‐bone graft	Capable of walking
							Replacement of loosening pedicle screws (L1, L4 and L5)	
							Correction of cage position	
							Extension of posterior instrumentation and fusion from T11 to S1	
18 y.o.	JUN/2013	T10 vertebral compression fracture	Decreased muscular strength;	Grade 3/5 muscular strength of bilateral iliopsoas, bilateral biceps femoris muscles and bilateral anterior tibialis	Vertebral compression fracture of T10		Revision:	
			Uncapable of completing physical activities				Removal of T11 pedicle screws	
							Extension of posterior instrumentation and fusion from T7 to T10	
19 y.o.	OCT/2014	Loosening pedicle screws	Lower limb weakness	Decreased muscular strength of lower limb muscles			Revision:	Muscular strength fully recovered
							Reinforcement of the broken pedicle walls by allo‐bone graft	
							Replacement of loosening pedicle screws (T7 and T8)	
							Removal of bony shells around T7 pedicle screws to decompress the spinal canal	
							Extension of posterior instrumentation and fusion from T4 to T10	
21 y.o.	OCT/2016	Migration of pedicle screws	Lower limb weakness	Limited motion of lumbar vertebra	Allo‐bones graft from T4 to T6 healed poorly		Revision:	Long‐term strict thoracolumbosacral orthosis protection
				Grade 3/5 muscular strength of bilateral iliopsoas, left and left quadriceps	Migrated pedicles screws of T4 compressed dual sac		Removal of T4, T5 and T6 pedicle screws	
				Bilateral Babinski sign (+), patellar and ankle clonus (+)	Central canal stenosis		Exploratory operation inside canals spinal from T4 to T6	
							Removal of medial pedicle walls of T4 and T5 to decompress the spinal canal	
							Extension of posterior instrumentation and fusion from T1 to T10	

**FIGURE 1:**
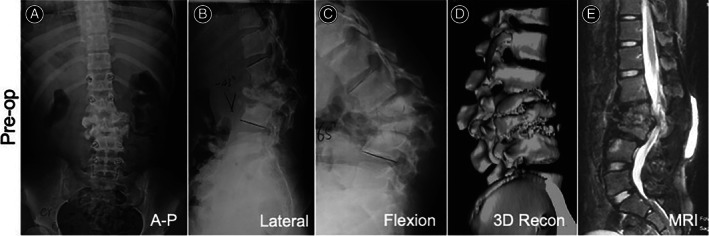
Initial radiological assessment showed lumbar kyphosis. (A) Anterior–posterior (A–P) view of the thoracic‐lumbar spine. (B) Kyphotic angle of 43° at the L2, L3 segment. (C) Kyphotic angle of 65° at flexion state; destructive appearance in centrums from L1 to L4. (D) Three‐dimensional reconstruction shows destruction of centrums. (E) MRI shows compression of the dual sac and stenosis of central canal. Figure 1A, B, E are cited from our previously published report on this case.^5^

In January 2010, the patient presented at our clinic due to lumbar kyphosis and frequent muscular weakness in lower limbs during walking. Physical examination noted increased motion of the lumbar vertebra during flexion‐extension movement. The patient's left lower limb measured 4 cm longer than the right. Charcot arthropathies were observed in both ankle joints. Muscle strength of the upper extremities were normal while muscle strength of right quadriceps and anterior tibialis was graded 4/5. The patient had normal muscle tension. Bilateral knee hyperreflexia was also noted. The other deep reflexes were normal. No pathological reflexes were induced. The patient had no pain perception although sensations to temperature, touch, position, two‐point discrimination, and vibratory sensibility remained intact. The patient had no history of bladder or bowel dysfunction. Laboratory examinations had no significant findings. X‐rays revealed a kyphotic angle of 43° at the L2, L3 segment (Figure 1A,B), and a kyphotic angle of 65° at her flexion state (Figure 1C). And there was a destructive appearance in centrums from L1 to L4 (Figure 1B–D). Sagittal magnetic resonance imaging (MRI) revealed dual sac compression and central canal stenosis (Figure 1E). Based on these findings, the patient was diagnosed with Charcot arthropathy, CIPA, lumbar kyphosis, and lumbar canal stenosis.

**FIGURE 2:**
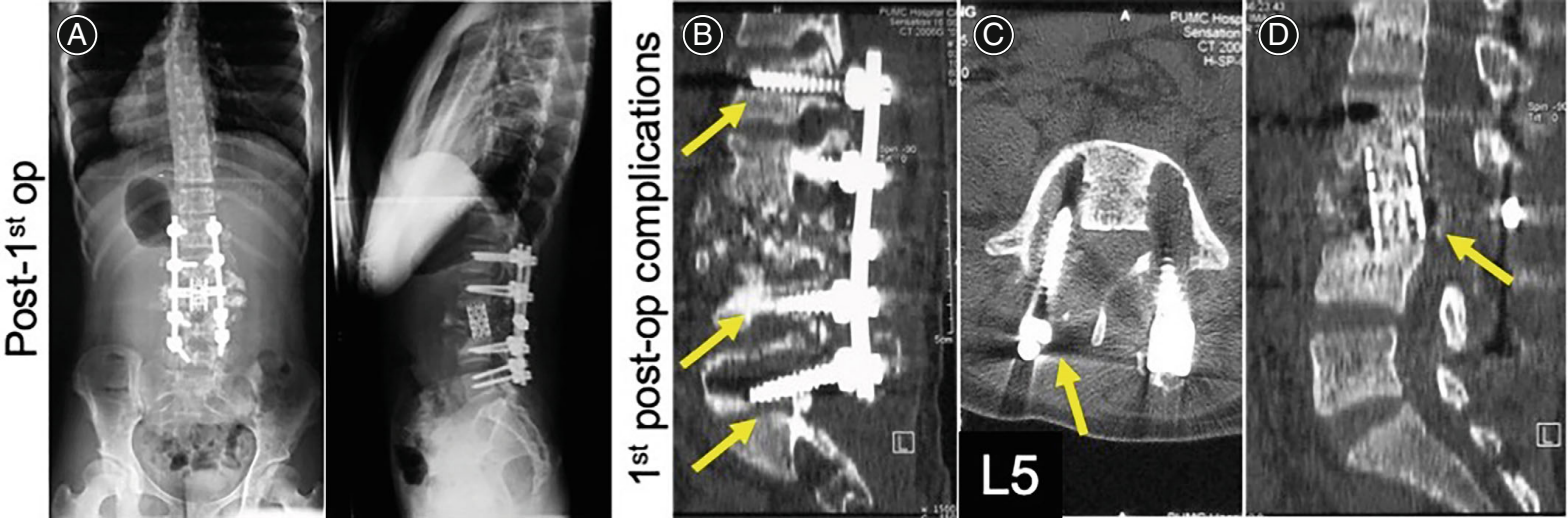
(A) Radiology, 3 months after the primary surgery: L2 and L3 corpectomy, instrumentation from T12 to L5, mesh cage. (B) Six months after the primary surgery, loosening pedicle screws of L1, L4, and L5. (C) MRI shows the loosening pedicle screws of L5. (D) Displaced mesh cage. Figure 2A is cited from our previously published report on this case.^5^

Here we gave the patient a posterior corpectomy of L2, L3, followed by reconstruction by mesh cage with autologous bone graft. Then we extended the instrumentation with pedicle screws from T12 to L5 and did a posterior‐lateral fusion with autograft bones. Surgery successfully corrected the patient's lumbar kyphosis. Complete remission of muscular strength was noted of her right quadriceps and anterior tibialis. Reassessment of this patient 3 months after the surgery showed radiographic evidence of solid bony healing as well as full and symmetric motor power in the lower extremities (Figure 2A).

**FIGURE 3:**
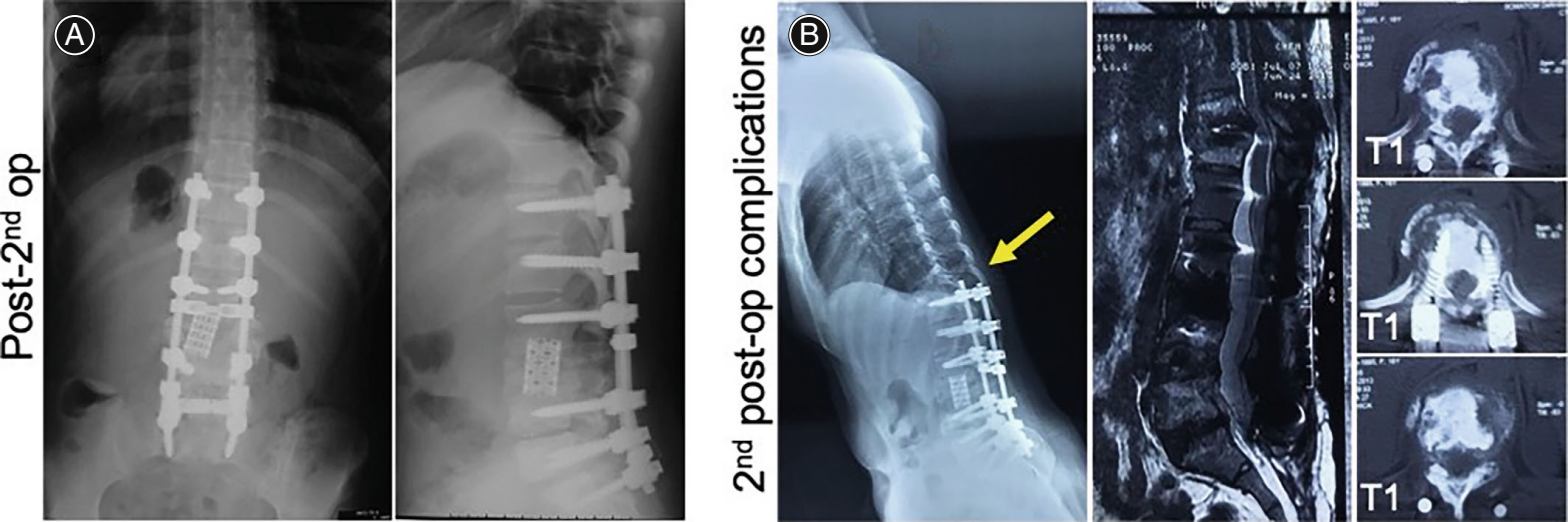
(A) Radiology, 1 year after the second surgery (i.e., 1st revision surgery): posterior interbody fusion of T12/L4, L4/L5, and L5/S1; mesh cage with corrected position; kyphotic angle was corrected to 0°. (B) Three years after the second surgery, T10‐compression fracture occurred.

**FIGURE 4:**
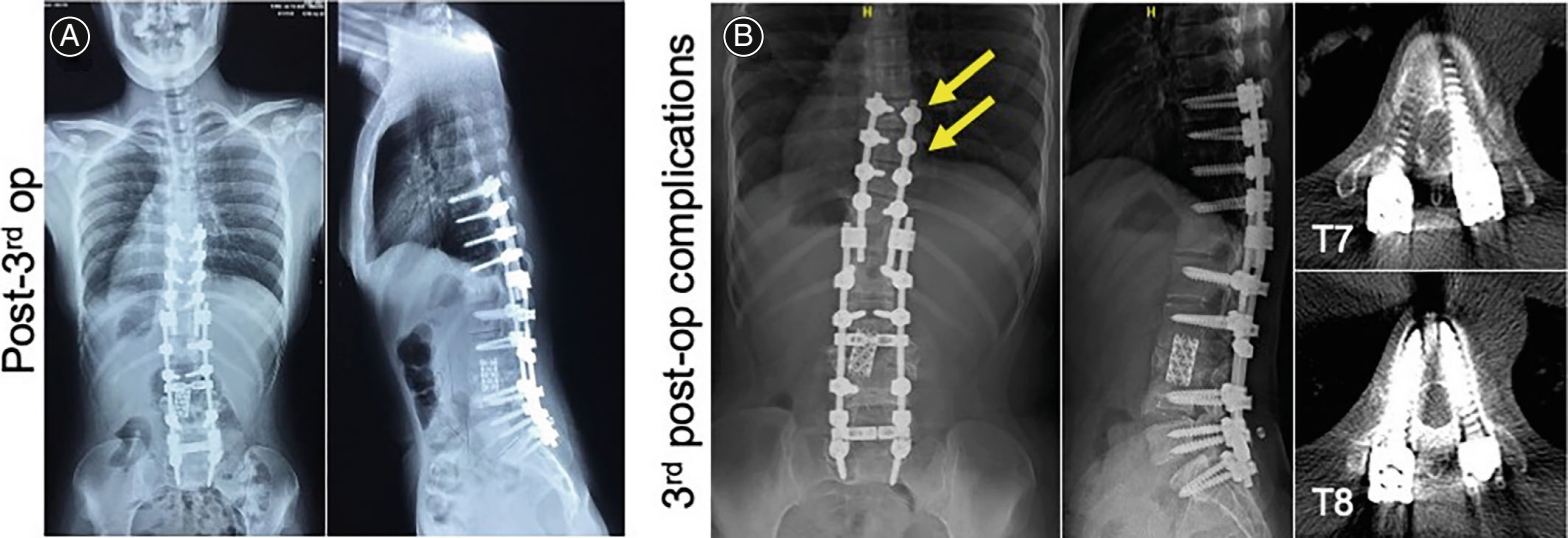
(A) Radiology, 2 months after the third surgery (i.e., second revision surgery): pedicle screws of T11 removed; posterior instrumentation extended from T7 to T10. (B) 1 year after the third surgery, loosening pedicle screws of T7 and T8 occurred.

**FIGURE 5:**
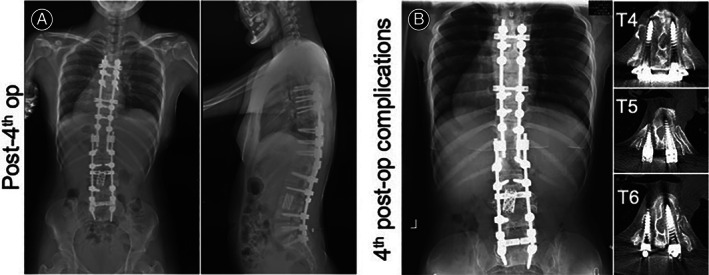
(A) Radiology, days after the fourth surgery (i.e., third revision surgery): posterior instrumentation extended from T4 to T10. (B) Two years after the fourth surgery, loosening pedicle screws of T4, T5, and T6, dural sac compression caused by T4 pedicle screw migration, and central canal stenosis were seen.

Unfortunately, her follow‐up visits in the following 4 years showed postoperative complications due to displacement of the mesh cage in 2010, loosening of pedicle screws in 2010 and 2014 and vertebral compression in 2013. These conditions caused the muscle weakness in her lower limbs before her revisit. In August 2010, 6 months after the primary surgery at our hospital to deal with the loosening pedicle screws of L1, L4, and L5 (Figure 2B, C) and displacement of the mesh cage (Figure 2D), we firstly reinforced the broken pedicle walls by allogeneic bone graft. Then we replaced the loosening pedicle screws with expansive pedicle screws, corrected the mesh cage's position, and then prolonged the posterior instrumentation from T11 to S1. To obtain a 360° solid fusion, we did a posterior interbody fusion at the interspaces of T12/ L1, L4/L5, and L5/S1 with allograft (Figure 3A). Posterior‐lateral fusion was performed using both allograft and autograft. (The initial assessment, primary surgery, and her first revision surgery were previously reported by us in 2013.^5^ In order for a more complete illustration, respective radiological images, that is, Figures 1A, B, E, and 2A, were cited from our previously published case report^5^). In June 2013, to deal with the T1‐compression fracture (Figure 3B), we removed the pedicle screws of T11 which were destabilized by the compression fracture of T10 and extended the posterior instrumentation from T7 to T10 with posterior interbody and posterior lateral fusion (Figure 4A). Even though the revision surgery was successful, her recovery was still unremarkable. And after the notion of loosening pedicle screws of T7 and T8 (Figure 4B), in October 2014, we placed them again at the same segments and prolonged the posterior instrumentation from T4 to T10 with posterior interbody and posterior lateral fusion (Figure 5A). Her postoperative recovery was uneventful. The patient's muscle strength recovered to 5/5. Daily physical activities including walking were restored. The X‐ray showed good lumbar alignment and solid lumbar spine fusion.

In October 2016, the patient again presented muscle weakness in the lower limbs when walking. Even standing was hard for the patient at her visit. Physical examination revealed limited motion of lumbar vertebra. The patient's left lower limb measured 8 cm longer than the right. Upper extremity strength remained normal. Meanwhile the strength of bilateral iliopsoas, left and left quadriceps was graded 3/5. In contrast, the strength of the patient's right quadriceps and right biceps femoris muscle was graded 2/5. Muscle tension of her lower limbs had increased abnormally. Her biceps tendon reflexes were normal, but the rest of deep reflexes were above normal. Babinski, Chaddock, and Oppenheim signs were induced bilaterally. Other pathological reflexes were not observed. The patient also presented symptoms of bilateral patellar clonus and ankle clonus. X‐rays revealed that the allogenic bones graft from T4 to T6 healed poorly, as well as compression of dural sac by the migration of pedicle screws of T4, which led to stenosis of the central canal (Figure 5B).

**FIGURE 6:**
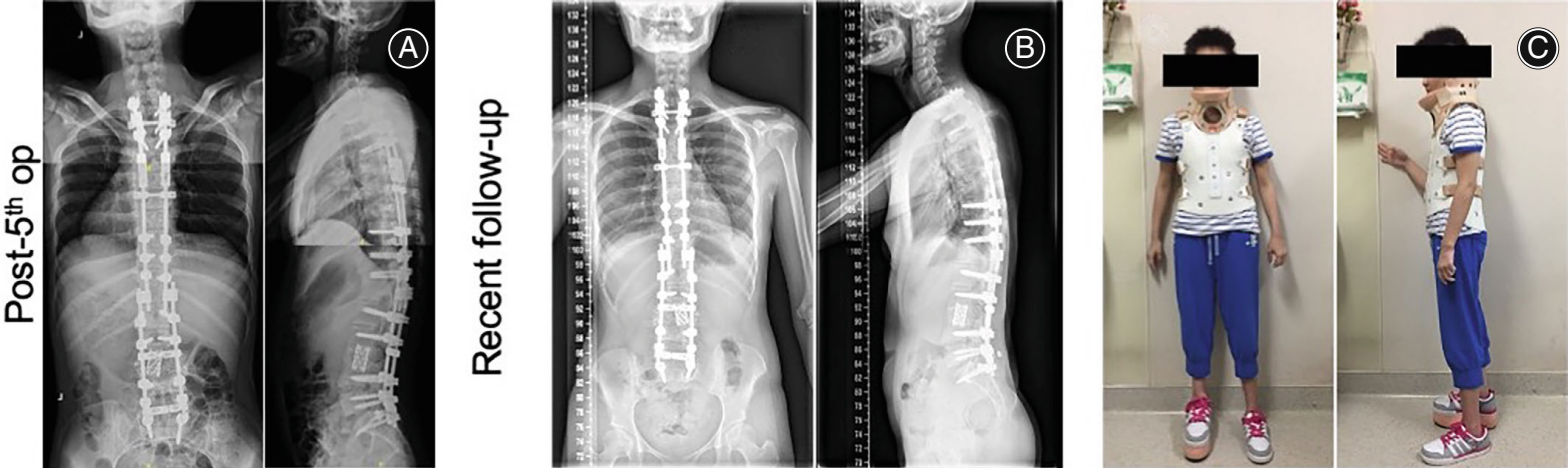
(A) Radiology, days after the fifth surgery (i.e., fourth revision surgery): posterior instrumentation extended from T1 to T10. (B and C) In a recent follow‐up visit, both clinical and radiological images showed good spine alignment and no loss of correction.

During hospitalization, revision surgery was again performed with a posterior approach. The pedicle screws of T4, T5, and T6 were removed. An exploratory operation on the spinal canal from T4 to T6 was performed. At the same time, the medial pedicle walls of T4 and T5, which compressed the dural sac, were removed to decompress the spinal canal. The posterior instrumentation was extended from T1 to T10. Posterior interbody fusion was performed at the interspaces of T1/ T2, T2/3, and T3/T4 with allograft and interbody cage (Figure 6A). Posterior lateral fusion was performed with allografts and autografts. The patient's postoperative recovery was still unremarkable. Strict long‐term thoracolumbosacral orthosis protection was prescribed for postoperative treatment. In a recent follow‐up visit, both her X‐ray and clinical images showed solid spine fusion and good spine alignment (Figure 6B, C).

## Discussion and Conclusions

As a rare condition, only a few hundred cases of CIPA have been reported world‐wide.^6^, ^7^ Here we present a case of a patient with typical clinical manifestations of Congenital Insensitivity to Pain with Anhidrosis (CIPA), including anhidrosis and insensitivity to pain, as well as bone lesions such as multiple fractures at an early age and Charcot arthropathy developed over time. Joint destruction and deformity are common complications in individuals with CIPA due to their inability to sense pain and respond to injury in a protective manner, which is defined as Charcot arthropathy. However, the patient's multiple fractures at an early age were likely due to her inability to feel pain and limited expression ability around the age of 3 years. As a result, her family did not give sufficient attention to the matter and failed to employ strict immobilization measures, leading to delayed fracture healing, limping, and ultimately multiple fractures. In the case of the femoral fracture caused by high‐energy injury, we propose that the child's lack of pain perception and protection awareness, combined with frequent childhood activity and falls from heights, may have contributed to this occurrence.

However, experiences with CIPA‐related Charcot arthropathy are still limited. From current opinion, corrective surgeries are recognized as the first line of treatment for such patients. However, the rareness of the disease imposes not only limitations to surgical strategies, but also management of peri‐operative complications and the long‐term restoration of physical mobility. Moreover, surgical procedures that correct Charcot spine caused by CIPA are fraught with technical challenges. Of the 16 cases of CIPA with Charcot spine that reviewed, 15 patients had undergone corrective surgery. The median follow‐up period of surgery cases that had follow‐ups is 2 years and only five cases received long‐term follow‐ups (>5 years). (Table 2) The primary complications that arose post‐surgery include hardware migration, adjacent segment disease, pedicle screw loosening, infection, pulmonary embolism, and paralysis. Besides, other clinical manifestations such as osteoporosis, reduced bone reconstruction, and recurrent factures cannot be explained by our current understanding of the disease's pathogenesis. Long‐term follow‐up with the patient reported herein might shed light on suitable surgical procedures. Clinical complications that arose post‐surgery with our patient as well as previous publications are also summarized to provide a better rationale for improving precautionary peri‐operative management in the future.

**TABLE 2:** Summary of previously reported cases of CIPA patients with Charcot spine on surgical approaches, clinical complications post‐surgery, and choices of revision surgeries

Ref.	Gender	Age of Dx (Yrs.)	Age of Manif. (Yrs.)	Affected Segments	Region	Follow‐up years	Anterior colum reconstruction	Surgical approach	Reconstruction strategy	Complications	Revision surgeries	Time intervals from primary surgery
[8]	F	0	25	T10‐11	T	N.A.	N.A.	Hemi‐laminectomy, posterior spinal fusion	none	Severe degree of paralysis unresolved		
[9]	M	17	17	T9‐11	T	N.A.	N.A.	N.A.	N.A.			
[10]	F	10	28	L1‐2	L	5	T	Posterior spinal fusion, delayed anterior fusion (6 mths)	autologous fibular strut	Proximal adjacent spondylolisthesis		
[11]	F	0	17	L3‐4	L	2	T	Corpectomy, delayed posterior spinal fusion (1 wk.)	autologous iliac crest strut			
[12]	F	0.5	12	L4‐5	L	1	F	Posterior decompression and fusion (L4‐S1)	none			
[13]	M	10	11	L1‐2	L	5	T	Anteroposterior spinal fusion	autologous rib and spinous process	Adjacent spondylolisthesis	Extended fixation to S1	5 years.
			16	L4‐5	L	2	F	Posterior spinal arthrodesis	none			
[14]	M	11	38	L5‐S1	L	N.A.	F	Posterior spinal fusion	none	Proximal adjacent spondylolisthesis	Extended fixation applied (unspecified segments)	Unspecified
[15]	M	1.5	23	L1‐2	L	2	T	Thoracotomy and anterior spinal fusion	Titanium cage	hardware migration	Extended fixation from T7 to L4	1 week.
[4]	F	N.A.	43	L2‐3, L4‐5	L	N.A.	N.A.	Posterior spinal fusion	none	Adjacent segment disease (L1‐2 dislocation); pulmonary embolism	Anteroposterior fusion	Unspecified
[5]	F	0	15	L2‐3	L	1.5	T	Corpectomy, posterior spinal fusion	autologous strut + mesh cage	hardware migration	Extended fixation from T11 to S1, interbody fusion	6 months
[16]	M	N.A.	16	Post‐trauma T6 paraplegia	T	~25	F	Posterior thoracic decompression, T2‐T9 fusion	Harrington rods			
			41	T10‐T11	T	2	T	Posterior spinal fusion (T2‐L1), Harrington rods removal, T10/11 anterior corpectomy and cage replacement	Titanium cage			
	M		11	T10	T	20	F	T8‐L1 posterior fusion	Unspecified	Worsening kyphotic deformity, adjacent segment disease (T12‐L1 lateral dislocation)	Posterior fusion, extended to L3, anterior reconstruction (T11‐L2)	18
[17]	M	3.5	27	thoracolumbar scoliosis	T‐L	N.A.	F	Posterior spinal fusion	Unspecified	decubitus lumbar ulcer, hardware infection	Hardware removal, immobilization with thoraco‐lumbar‐sacral orthosis	Unspecified
	M	2.5	24	thoracolumbar kyphoscoliosis	T‐L	N.A.	F	Posterior spinal fusion	Unspecified	decubitus lumbar ulcer, chronic osteomyelitis; paraplegia	Hardware removal	Unspecified
[2]	F	5	28	L5	L	1.5	T	Posterior spinal fusion	cage	L2 pedicle screws, suspicious for hardware loosening		
	M	N.A.	32	L4，post‐op of L1‐3 fusion	L	5	T	Posterior spinal fusion	cage + mesh cage	none		
			37	C5/6	C	N.A.	N.A.	Anterior spinal fusion	ACDF	none		

### Hardware Migration

Among the 14 patients that underwent surgery, seven patients received anterior reconstruction and fusion (spinal fusion, bone graft in vertebral lesions, titanium cages with bone grafts).^2^, ^5^, ^10^, ^11^, ^13^, ^15^ Removal of damaged vertebrae and subsequent reconstruction by mesh cage with extended height (over the height of one vertebra) increased the likelihood of migration. Cage dislocation occurred shortly (1 week and 6 months, respectively) after the corpectomy of vertebral lesions in two patients.^2^, ^5^ The other three patients that underwent partial removal of the damaged vertebrae and autologous bone graft from either fibula, ilium, or rib achieved satisfactory spinal fusion and stability. Hardware migration was also not observed in patients that were implanted with intervertebral cage or mesh cage with bone graft.^2^

In conclusion, the broad removal of spinal lesions and reconstruction with over‐height mesh cage appears to be correlated with an increased risk of hardware migration and are thus not recommended.

### Adjacent Segment Diseases

The patient reported herein as well as six other patients in previous reports developed adjacent segment diseases (ASDs) proximal or distal to the bone graft fusion sites, typically after the fusion had stabilized.^2^, ^4^, ^5^, ^10^, ^13^, ^14^, ^15^ Staudt et al. proposed that fixation, beginning at the proximal end of the thoracolumbar junction and extending toward the distal end of S1, is reliable for dealing with thoracolumbar lesions.^2^ However, ASDs of T10 still occurred after fixation from T11 to S1. The underlying causes of ASDs are yet to be determined. Apart from the fact that CIPA itself could again cause Charcot spine post‐surgery, other possible contributing factors include disturbed intervertebral mechanical characteristics and altered anatomical structure.

Alterations of intervertebral pressure distribution and instability of adjacent segments are unavoidable post‐surgery. Long‐segment fixation might thus increase the stability and reduce the risk of adjacent segment diseases. However, unnecessarily fixing additional segments could further reduce the patients' quality of living. Determining the proper segments to fix remains a challenge to be addressed.

### Pedicle Screw Loosening

Loosening of bilateral pedicle screws in the fused segments of CIPA patients who had no overt symptoms has been previously reported.^2^ However, our patient had successive loosening of the pedicle screws. The loosened screws gradually migrated inward and compressed the spinal canal, causing severe incomplete paralysis. Consequently, revision surgery was conducted to relocate the screws, decompress the spinal canal, and extend the fixation segments to T1.

Pedicle screw loosening could be related to altered vertebral bone structure. Osteoporosis and delayed bone reconstruction might be related to CIPA and could potentially lead to loosening of the pedicle screws.

### Infection

Kayani et al. reported two patients who developed decubitus and implant infection.^17^ The internal fixation hardware was eventually removed to relief the infection. Staudt et al. reported one case with soft‐tissue infection post‐surgery.^2^ After long‐term administration of broad‐spectrum antibiotics and vacuum‐assisted wound closure, the infection was restricted.

On one hand, the risk of peri‐operative infection in patients receiving spinal surgeries can be high. On the other, patients also risk immunodeficiency brought about by defective NGF/NTRK1 signaling in CIPA patients. An early study demonstrated that a mutation to NTRK1 could lead to a defect in B cells due to failure of cytoskeleton formation and MAP kinase activation.^18^

Our patient did not suffer from peri‐operative infection despite having received five surgical operations. In this regard, the prevention and management of infectious diseases such as decubitus and implant infection in CIPA patients should receive additional attention.

### Paralysis

The early identification of spinal injuries arising from post‐internal fixation surgeries, including hardware migration and adjacent segment disease, in CIPA patients can be challenging due to their inability to sense pain. In this case, ASD was not noticed until the occurrence of incomplete paralysis. Therefore, thorough and frequent physical examinations during follow‐up visits will be ideal for preventing paralysis in patients with high risks of spinal injuries post‐operation.

### NTRK1 Deficiency and its Impact on Bone Mineral Metabolism

Repeated implant failure and compressive vertebral fracture that occurred in our patient suggest that CIPA patients with Charcot spine could suffer from impaired bone formation after undergoing corrective surgery, in turn extending the recovery period post‐surgery. Meanwhile, radiological examinations revealed severe osteoporosis in vertebral and multiple limb bones, which is likely to be another direct clinical complication of CIPA. Lack of exercise and sun exposure due to immobility caused by the disease may also result in impaired bone construction and vitamin D synthesis, increasing the risk of osteoporosis. Moreover, impaired signaling via NGF/NTRK1 itself might be the direct cause of defective bone healing.

The peripheral sensory nerves projecting from nociceptive dorsal ganglions (DRGs) are mainly comprised of myelinated Aα/β‐ and Aδ‐fibers, and unmyelinated C‐fibers.^19^ Mineralized bones and bone marrow are innervated by Aδ‐fibers that express high levels of NGF and its receptor NTRK1 (TrkA). The NGF/NTRK1 signaling pathway is crucial in the maintenance of neuron survival and differentiation.^20^ Blockade of the signaling pathway via inhibitors or antibodies have already been exploited as strategies for managing pain.^20^, ^21^ Also, genetic mutations that abrogate NGF/NTRK1 signaling, which in this case is caused by a missense mutation of NTRK1, would cause neurodegeneration of afferent Aδ‐fibers and C‐fibers and further loss of sensation that could potentially lead to neuropathic Charcot arthropathies. In the meantime, studies with animal models have shown that NGF/NTRK1 signaling is critical for vascularization, primary and secondary ossification during the development of endochondral bones. Disturbance of the pathway results in impaired bone growth, reduced bone mineral density, and also reduced bone volume.^22^ However, evidence supporting involvement of the NGF/NTRK1 signaling pathway in bone recovery post‐trauma/surgery remains limited and controversial. Studies on a murine fracture and pin placement model have shown that inhibition of either NGF or NTRK1 is associated with increased callus size at the fracture site, suggesting that the disturbance of NGF/NTRK1 signaling dampens biomechanical properties. Notwithstanding, gross bone bridging in these two studies did not seem to be significantly different between the inhibited and untreated groups.^23^, ^24^ In contrast, another recent study claimed that the administration of neutralizing antibodies to NTRK1 in fractured mice did not interfere with the bone healing process.^25^ As for human research, the mutation in NTRK1 was determined by whole‐exome sequencing (WES) analysis to be related to osteogenesis imperfecta.^26^ Analysis of ex vivo osteoblasts and their mineralization ability have implicated several critical pathways in bone metabolism, including NTRK1 as a player in the nuclear factor kappa‐light‐chain‐enhancer of activated B cells (NF‐κB) signaling.^27^ Hence, both animal and human studies show that NGF/NTRK1 signaling contributes to the maintenance of bone mineral density and prevents osteoporosis in both a nerve‐ and metabolism‐dependent manner.

### Perioperative Management

The optimal treatment regimen for CIPA patients after surgery is still a matter of debate. Considering the delayed bone healing process and increased risk of osteoporosis, supplementation with vitamin D and calcium supplement could be of help. Extra caution should be taken when administrating bisphosphonates, as such treatments have been shown to lower the turnover of bones and increase the risk of new fractures.^28^, ^29^ Teriparatide, an effective anabolic agent, might be incorporated into the regimen to induce bone formation.^30^ However, the administration of teriparatide to younger patients is associated with the risk of osteosarcoma. The benefits and risks following the administration of teriparatide should be carefully considered. Fever in patients, especially post‐surgery, can be regarded as a clinical complication arising from malfunction of the sensory circuit.^31^ However, laboratory test results should be carefully interpreted to rule out possibilities of infections due to their immune predisposition.

### Concluding Remarks

In this case report, a patient had typical clinical manifestations of CIPA, including loss of sensation of pain, anhidrosis, and intellectual deficiency. The patient also developed severe Charcot spine and consequently received several surgeries to fix the spine from T1 to S1. The clinical complications that arose include ASDs and incomplete paralysis secondary to pedicle screw loosening and migration, all of which were resolved by multiple revision surgeries.

In summary, based on our case and previous reports, we do not recommend large‐scale removal of damaged vertebrae and subsequent reconstruction for CIPA patients with Charcot spine as it might increase the risk of hardware migration. With respect to fixation, we recommend a 360° long‐segment fusion to reduce the risk of ASDs. Nevertheless, complications including adjacent segment diseases and pedicle screw loosening may still arise. As such, frequent follow‐up visits and thorough physical examinations post‐surgery should assist in the early identification of these complications before the development of severe neurological lesions. Lastly, careful nursing, proper rehabilitation exercises, and treatments that improve bone mineral metabolism will also be critical to the peri‐operative management of CIPA patients, which will improve surgical recovery and quality of life.

## Author Contributions

Siyi Cai and Yuhao Jiao analyzed and interpreted the patient data. Yuhao Jiao wrote the case presentation, did the literature review, and wrote the discussion and conclusions. Ye Tian performed surgeries for this patient. Ye Tian and Siyi Cai are experienced orthopaedic surgeons. Siyi Cai supervised the case report and literature review. All authors read and approved the final manuscript for publication.

## Conflict of Interest Statement

The authors declare that they have no competing interests.

## Ethics Statement

All the clinical data to publish on individual patients has been approved by the institutional ethics committee of Peking Union Medical College Hospital. Written informed consent was obtained from the patients for publication of this case report and any accompanying images.

## Data Availability

Unpublished patient data including laboratory studies and medical imaging generated or analyzed during this study are available from the corresponding author on reasonable request.
